# Polymorphisms in the P2X7 receptor, and differential expression of Toll-like receptor-mediated cytokines and defensins, in a Canadian Indigenous group

**DOI:** 10.1038/s41598-019-50596-0

**Published:** 2019-10-02

**Authors:** Catlin Semple, Ka-Yee Grace Choi, Andrea Kroeker, Lizette Denechezhe, Pamela Orr, Neeloffer Mookherjee, Linda Larcombe

**Affiliations:** 1 Department of Medical Microbiology and Infectious Disease, Max Rady Faculty of Medicine, University of Manitoba, Winnipeg, MB Canada; 2 Department of Internal Medicine, Max Rady Faculty of Medicine, University of Manitoba, Winnipeg, MB Canada; 3 Northlands Denesuline First Nation, Lac Brochet, MB Canada; 4 Department of Community Health Sciences, Max Rady Faculty of Medicine, University of Manitoba, Winnipeg, MB Canada; 5 Department of Immunology, Max Rady Faculty of Medicine, University of Manitoba, Winnipeg, MB Canada

**Keywords:** Medical research, Risk factors

## Abstract

Canadian Indigenous peoples (First Nations and Inuit) exhibit a high burden of infectious diseases including tuberculosis influenced by societal factors, and biological determinants. Toll-like receptor (TLR)-mediated innate immune responses are the first line of defence against infections. We examined the production of a panel of 30 cytokines in peripheral blood-derived mononuclear cells (PBMC) isolated from Indigenous and non-Indigenous participants, following stimulation with five different TLR ligands. The levels of TLR-induced pro-inflammatory cytokines such as IL-12/23p40, IL-16, and IFN-γ, and chemokines (MCP-4, MDC and eotaxin) were different between Indigenous compared to non-Indigenous participants. Antimicrobial cationic host defence peptides (CHDP) induced by TLR activation are critical for resolution of infections and modulate the TLR-to-NFκB pathway to alter downstream cytokine responses. Therefore, we examined the expression of human CHDP defensins and cathelicidin in PBMC. mRNA expression of genes encoding for *def-A1* and *def-B1* were significantly higher following stimulation with TLR ligands in Indigenous compared to non-Indigenous participants. The purinergic receptor P2X7 known to be activated by ATP released following TLR stimulation, is a receptor for CHDP. Therefore, we further examined single nucleotide polymorphisms (SNP) in P2X7. Indigenous participants had a significantly higher percentage of a P2X7 SNP which is associated with reduced function and lower ability to clear infections. These results suggest that a higher frequency of non-functional P2X7 receptors may influence the activity of downstream immune mediators required for resolution of infections such as pro-inflammatory cytokines and CHDP defensins, thus contributing to higher burden of infections in Indigenous population.

## Introduction

The Canadian First Nations and Inuit groups experience a higher burden of infectious and chronic diseases compared to non-indigenous populations. Among infectious diseases, the incidence of tuberculosis (TB) is particularly high. Although Indigenous peoples comprise 4% of the Canadian population, they account for 17% of all reported cases in Canada^1^. In 2014, the incidence rate of active TB in Canadian-born non-Indigenous population reported was 0.7 per 100,000 people, while the incidence of active TB was 28.8 per 100,000 people among Canadian-born indigenous population^2,3^.

The determinants of higher infection burden among Canada’s indigenous peoples are predominantly socioeconomic and political, including the historical legacy of colonialism and stigma, poverty, housing and nutrition, and inadequacies in the quality and availability of care^4–8^. Recent studies have also identified biological factors that may influence disease susceptibility among indigenous^9–12^. These include innate immune responses required to resolve bacterial and viral infections, and genetic variation e.g. single nucleotide polymorphisms (SNP) in critical immunity-related genes^9,11,13^.

Toll-like receptors (TLR) are critical components of the anti-infective immune response. These receptors recognize pathogen-associated molecular patterns (PAMP) triggering downstream innate immune responses against pathogenic challenge such as bacterial and viral infections^14–17^. TLRs are either expressed on the surface of immune cells (i.e. extracellular such as TLR 1/2, 4, 5 and 6) or expressed inside the cell (i.e. endogenous TLR such as TLRs 3, 7, 8 and 9). The overall repertoire and expression orientation of TLR facilitate detection of both extracellular and intracellular PAMP. Each member of the TLR family recognizes distinct PAMP; for example TLR1/2 and TLR6 interact with pathogen-associated lipoproteins, TLR3 ligand are typically double-stranded RNA, TLR4 recognizes bacterial lipopolysaccharide (LPS), TLR5 interacts with bacterial flagellin, TLR7 and TLR8 recognize intracellular single-stranded RNA such as viral ligands, and TLR9 recognizes bacterial CpG DNA^18–24^. Following interaction with its respective ligands, TLR initiate a series of signaling cascades resulting in the production of innate immune response elements such as cytokines, chemokines and cationic host defence peptides (CHDP)^25–27^. These peptides are immune effector molecules that facilitate recruitment of immune cells to the site of infections, and orchestrate initiation and differentiation of adaptive immune responses, thus CHDP play an important role in the effective resolution of infections. Previous studies have demonstrated that impaired TLR functions result in increased susceptibility to infections and inadequate control of pathogens^28–31^.

We have previously shown that the expression of TLR1/2-mediated immune response elements induced in the presence of a *Mycobacterium tuberculosis* (MTB)-related lipoprotein in macrophages are differentially regulated in a Canadian First Nation (Dene) population, compared to a Canadian non-Indigenous cohort^11^. This included the expression of specific cytokines and a human CHDP cathelicidin LL-37. Expanding upon our previous study^11^, we have comprehensively evaluated various TLR ligand (TLRL) mediated downstream immune responses in peripheral blood-derived mononuclear cells (PBMC) obtained from obtained from Dene, Inuit and non-Indigenous Canadian participants in this study. A panel of TLRL representing both extracellular and intracellular infectious agents were selected as stimulants in this study. We hypothesized that TLR-mediated innate immune responses such as specific cytokine and CHDP expression required for antimicrobial functions may be differentially expressed within the Canadian Dene and Inuit populations, which may be a contributing factor for consequent high burden of infections. In this study, we demonstrate that certain cytokines and CHDP such as defensins are differentially expressed in responses to TLRL in PBMC isolated from the Dene and Inuit, compared to non-Indigenous participants. We further show that the Dene exhibit higher frequency of specific single nucleotide polymorphisms (SNP) in the purinergic P2X7 receptor compared to non-Indigenous. The P2X7 receptor is expressed by immune cells, is a known receptor for CHDP^31,32^ and plays an important role in innate and adaptive immunity^33^. Previous studies have established a clear link between TLR activation and P2X7-mediated functions; for example, TLR activation results in adenosine triphosphate (ATP) release which in turn activates the P2X7 receptor^34,35^. Activation of the P2X7 receptor leads to production of reactive oxygen species and inflammatory mediators from phagocytic cells, responses required for clearance of pathogens^33,35^. Furthermore, activation of several innate immune genes downstream of the TLR-to-NF-κB pathway is also dependent on the P2X7 receptor function^33^. Therefore, results from this study suggest that SNP in P2X7 receptor, along with differential expression of TLR-mediated responses such as defensins, which play a critical role in antimicrobial functions, may contribute to higher burden of infections in Dene and Inuit peoples.

## Results

**Figure 1:**
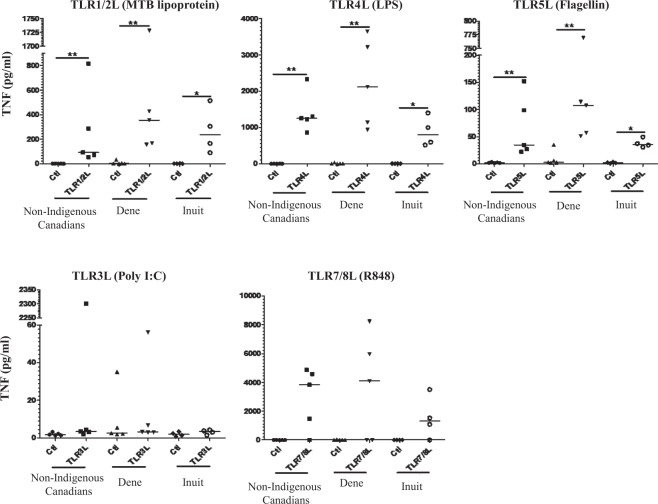
TLR ligands-induced pro-inflammatory cytokine TNF-α production in PBMC isolated from Indigenous and Non-Indigenous participants. Human PBMC isolated from the study participants were stimulated with different TLR ligands; MTB lipoprotein (100 ng/ml), LPS (100 ng/ml), Flagellin (100 ng/ml) or R848 (1 μg/ml). Tissue culture supernatants were examined for the production by ELISA for pro-inflammatory cytokine TNF after 24 hr. Each dot represents an independent experiment from PBMC isolated independent participants. Mann-Whitney U test was used for statistical analyses (*p < 0.05, **p,0.005).

**Figure 2:**
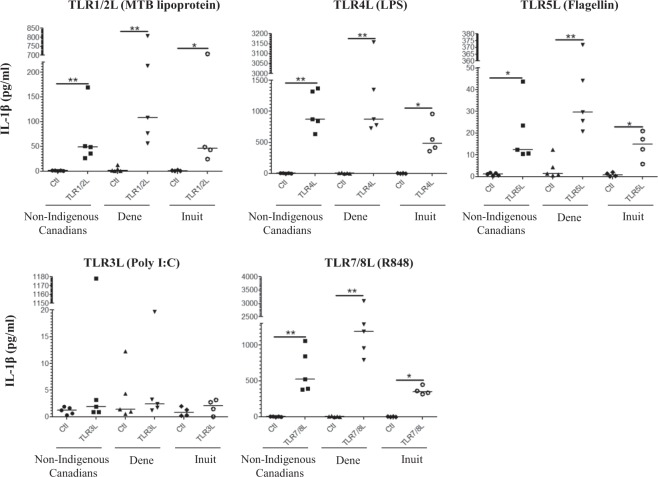
TLR ligands-induced pro-inflammatory cytokine IL-1β production in PBMC isolated from Indigenous and Non-Indigenous participants. Human PBMC isolated from the study participants were stimulated with different TLR ligands; MTB lipoprotein (100 ng/ml), LPS (100 ng/ml), Flagellin (100 ng/ml) or R848 (1 μg/ml). Tissue culture supernatants were examined for the production by ELISA for pro-inflammatory cytokine IL-1β after 24 hr. Each dot represents an independent experiment from PBMC isolated independent participants. Mann-Whitney U test was used for statistical analyses (*p < 0.05, **p,0.005).

**Figure 3:**
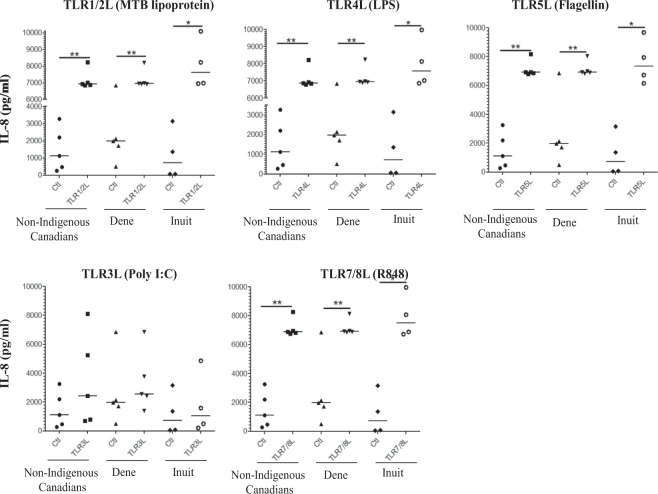
TLR ligands-induced neutrophil chemokine IL-8 production in PBMC isolated from Indigenous and Non-Indigenous participants. Human PBMC isolated from the study participants were stimulated with different TLR ligands; MTB lipoprotein (100 ng/ml), LPS (100 ng/ml), Flagellin (100 ng/ml) or R848 (1 μg/ml). Tissue culture supernatants were examined for the production by ELISA for chemokine IL-8 after 24 hr. Each dot represents an independent experiment from PBMC isolated independent participants. Mann-Whitney U test was used for statistical analyses (*p < 0.05, **p,0.005).

Innate immune responses to infectious challenge are primarily orchestrated by the interaction of specific pathogen-associated molecular ligands with various TLR resulting in the downstream production of pro-inflammatory cytokines and chemokines^36–39^. Therefore, we monitored the production of pro-inflammatory cytokines (TNFα and IL-1β) and that of the neutrophil chemokine IL-8, following stimulation of PBMC isolated from study participants with various TLRL. Exogenous TLRL, MTB lipoprotein (TLR1/2L), LPS (TLR4L) and flagellin (TLR5L) simulating bacterial ligands, as well as the endogenous TLRL such as R848 (TLR7/8L) and poly I:C (TLR3L) simulating viral challenge, significantly induced the production of pro-inflammatory cytokines TNFα (Fig. 1) and IL-1β (Fig. 2) and neutrophil chemokine IL-8 (Fig. 3), in PBMC obtained from all three groups i.e. non-Indigenous, Dene and Inuit. There was no significant difference between the levels of TNFα, IL-1β and IL-8 in PBMC isolated from non-Indigenous, Dene or Inuit (Figs 1–3 respectively).

### TLRL-induced biosignature in PBMC is different in Dene participants

**Figure 4:**
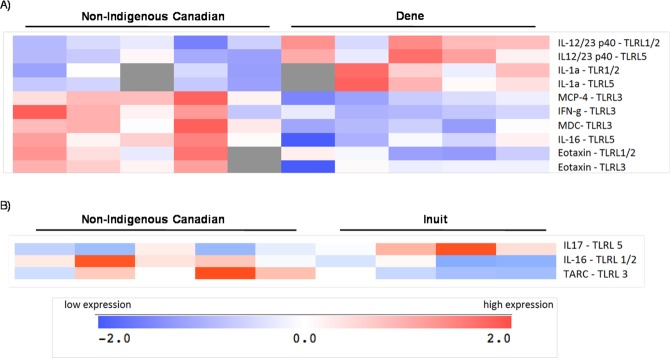
Differential production of cytokines in response to specific TLR-ligands in PBMC isolated from Indigenous and Non-Indigenous participants. Human PBMC isolated from the study participants were stimulated with different TLR ligands and the tissue culture supernatants were examined for the production of 30 different cytokines using multiplex MesoScale Discovery platform. Cytokine concentrations were normalized with each participant’s own internal control (unstimulated cells). The Multi-Experiment Viewer analysis tool was used to compare the profile of protein production in samples from (**A**) Non-Indigenous Canadians and Dene, and (**B**) Non-Indigenous Canadians and Inuit. Statistical analyses was performed using the Welch approximation, and statistical significance was defined as p < 0.05. Cytokines shown are those that were significantly different between the groups compared.

To elucidate a more comprehensive effect of TLR-mediated cytokine production, we examined the production of a panel of 30 different cytokines and chemokines using a multiplex MesoScale Discovery platform, in the tissue culture (TC) supernatants obtained following stimulation of PBMC with different TLRL, and compared these responses between the three groups (non-Indigenous, Dene and Inuit). PBMC were stimulated with various TLRL; MTB lipoprotein, LPS, flagellin, poly I:C and R848, and TC supernatants were monitored for the production of a panel of 30 analytes (as described in methods) after 24 h. Results obtained were normalized with each participant’s own internal control (unstimulated cells). Using the Multi-Experiment Viewer analysis tool we compared the cumulative profile of protein production in samples from the three study groups; Dene, Inuit and non-Indigenous. We identified 12 cytokines that were significantly different (p < 0.05) between the Dene and non-Indigenous population; IL-12/23 p40 and IL-1a (following stimulation with TLR1/2L MTB lipoprotein and TLR5 ligand flagellin), MCP-4, IFN-γ and MDC (following stimulation with TLR3 ligand poly I:C), flagellin-induced IL-16 (with flagellin stimulation) and eotaxin (with MTB lipoprotein and poly I:C stimulation) (Fig. 4A). There were only three proteins that were significantly different (p < 0.05) between the Inuit and non-Indigenous populations; flagellin-induced IL-17, MTB lipoprotein-induced IL-16 and poly I:C-induced TARC (Fig. 4B). These results suggested that TLR1/2, TLR3 and TLR5 ligands-induced cytokines were differentially expressed in the Indigenous (Dene or Inuit) compared to the non-Indigenous participants.

### TLRL-induced defensins are differentially expressed in an Indigenous population

**Figure 5:**
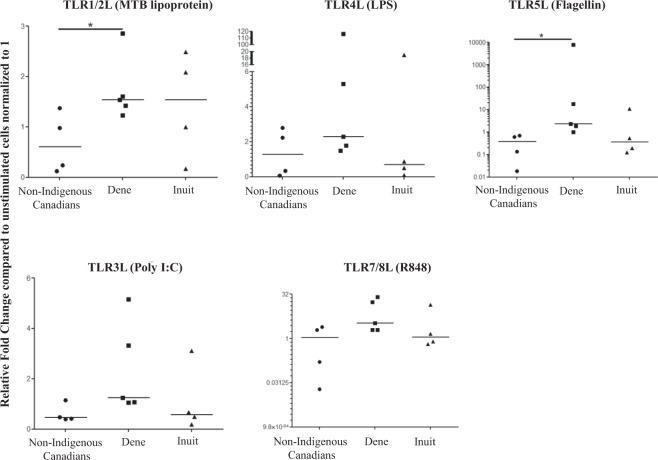
mRNA expression of gene *defA-1* encoding for α-defensin in PBMC isolated from Indigenous and Non-Indigenous participants. Human PBMC isolated from the study participants were stimulated with different TLR ligands; MTB lipoprotein (100 ng/ml), LPS (100 ng/ml), Flagellin (100 ng/ml) or R848 (1 μg/ml), for 6 hr. mRNA isolated was examined for the expression of *defA-1* by quantitative real-time PCR. Relative fold changes were calculated compared to the expression in the unstimulated cells normalized to 1, using the standard ΔΔCt method, after normalization with 18sRNA expression. Each dot represents an independent experiment from PBMC isolated independent participants. Mann-Whitney U test was used for statistical analyses (*p < 0.05, **p,0.005).

**Figure 6:**
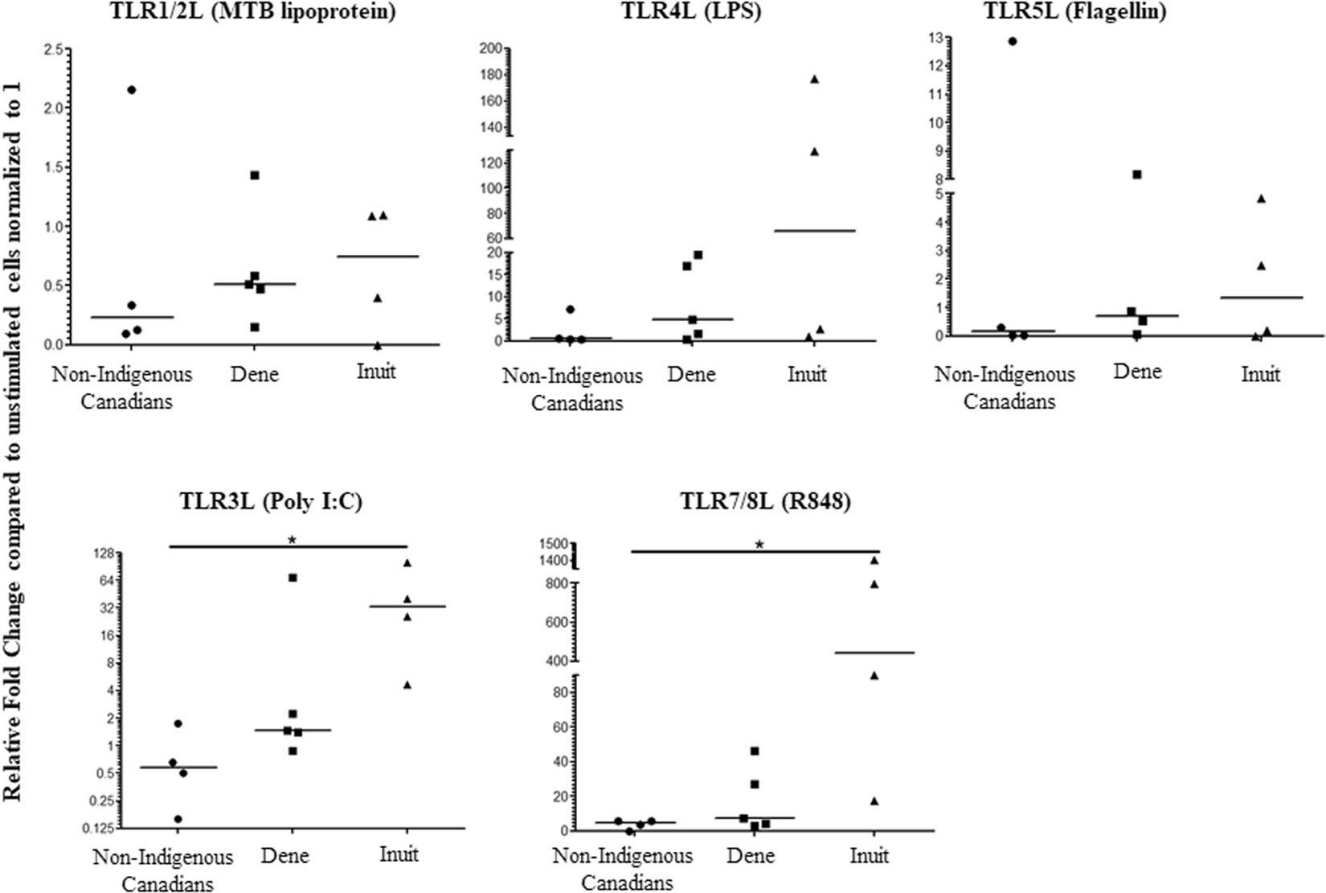
mRNA expression of gene *defB-1* encoding for  β-defensin in PBMC isolated from Indigenous and Non-Indigenous participants. Human PBMC isolated from the study participants were stimulated with different TLR ligands; MTB lipoprotein (100 ng/ml), LPS (100 ng/ml), Flagellin (100 ng/ml) or R848 (1 μg/ml), for 6 hr. mRNA isolated was examined for the expression of *defB-1* by quantitative real-time PCR. Relative fold changes were calculated compared to the expression in the unstimulated cells normalized to 1, using the standard ΔΔCt method, after normalization with 18sRNA expression. Each dot represents an independent experiment from PBMC isolated independent participants. Mann-Whitney U test was used for statistical analyses (*p < 0.05, **p,0.005).

Human CHDP such as α-defensin, β-defensin and cathelicidin LL-37 are critical immunomodulatory molecules known to regulate the production of cytokines and chemokines in PBMC following TLR stimulation. These peptides are also potent antimicrobial agents. In addition, TLR activation induces the production of CHDP. As the TLR-CHDP axis plays a critical role in control of infections, we further monitored the mRNA expression profile of three human CHDP in PBMC following stimulation with the different TLRL. RNA was isolated from PBMC following stimulation with various TLRL- (MTB lipoprotein, LPS, flagellin, poly I:C and R848) after 6 h. mRNA expression of genes encoding for CHDP α-defensin (*defA-1*), β-defensin (*defB-1*) and cathelicidin LL-37 (*camp*) was monitored using qRT-PCR. Bacterial ligands MTB lipoprotein and flagellin – induced *defA-1* mRNA expression was significantly higher in PBMC isolated from Dene compared to that from non-Indigenous participants (Fig. 5). Whereas, viral ligand poly I:C and R848-induced d*efB-1* mRNA expression was significantly higher in PBMC isolated from Inuit compared to that in non-Indigenous participants (Fig. 6). There were no statistically significant differences in mRNA expression of either *defA-1* or *defB-1* following TLR ligand stimulation in PBMC isolated from Dene and Inuit participants (Figs 5 and 6). There were no significant differences in TLR-mediated *camp* mRNA expression between the three groups (Supplementary Fig. S1). We further examined the concentration of CHDP cathelicidin LL-37, and defensins hBD2 and HNP1-34, in TC supernatants obtained from PBMC stimulated with the different TLRL after 24 hours, by ELISA. There was no significant difference in the concentration of these peptides in the TC supernatants between any of the groups in this study (Supplementary Fig. S2).

### Human purinergic receptor P2X7 SNP

Both α- and β-defensin mediated regulation of TLR-induced cytokine expression involves the ATP-gated channel receptor P2X7^32,40^. TLR activation-mediated ATP release activates the P2X7 receptor. The P2X7 receptor is expressed by immune cells and plays a critical role in immune responses to pathogens^33^. Furthermore, P2X7 gene polymorphisms are associated with the susceptibility to infectious diseases such as tuberculosis^41^. Therefore, we evaluated SNPs in the P2X7 gene. Table 1 details the allele and genotypes frequencies for the Indigenous population, which was significantly different from the non-Indigenous population. The Dene population had significantly higher frequency of C allele and AC and CC genotypes than the non-Indigenous group. The Inuit population also demonstrated higher frequency of C allele and AC genotypes compared to the non-Indigenous population. These results demonstrated that there was a higher frequency of C allele in P2X7 gene polymorphisms among the Indigenous population compared to the non-Indigenous. We expanded this analysis with 114 self-identified Dene individuals and 99 self-identified non-Indigenous individuals (Table 2). Hardy-Weinberg Equilibrium was assessed for the populations in the genetic study. Neither the Dene (*X*^2^ = 43.4, p < 0.005) or the non-Indigenous (*X*^2^ = 4.8, p = 0.027) population were in Hardy-Weinberg Equilibrium for the P2X7 receptor SNP, which might raise some questions about the randomness of the samples collected and/or the degree of selective pressure on this SNP. Nevertheless, similar to our results in the smaller cohort (Table 1), the Dene population showed a statistically higher frequency of C allele and CC genotype compared to non-Indigenous population when the analysis was expanded using a larger study cohort (Table 2).

**Table 1:** P2X7 receptor (rs3751143) allele and genotype frequencies.

P2X7 receptor rs3751143		Allele Frequency %		Genotype Frequency %			P Value
Populations		A	C	AA	AC	CC	
Non-Indigenous	Caucasian (n = 7)	86	14	71	29	0	p < 0.005
Indigenous	Dene (n = 5)	50	50	17	67	17	
	Inuit (n = 5)	80	20	60	40	0	p < 0.005

**Table 2:** P2X7 receptor (rs3751143) allele and genotype frequencies for Dene and non-Indigenous larger cohort.

P2X7 receptor rs3751143	Allele Frequency %		Genotype Frequency %			P Value
	A	C	AA	AC	CC	
Non-Indigenous (n = 99)	85	17	72	22	6	p < 0.005
Dene (n = 114)	47	53	47	20	42	

## Discussion

Intervention at the level of the socioeconomic determinants of infectious diseases among Canada’s indigenous peoples is of primary importance in the effort to reduce the overall burden of illness and the disparities that exist in disease burden. However, an understanding of the biologic determinants is also important in the development of therapeutics, including effective vaccines and medications. Despite the importance of TLR-mediated signalling in resolution of infections, a comprehensive study evaluating the differential effects of different TLRL-induced responses in leukocytes from Canadian Indigenous (Dene and Inuit) and non-Indigenous populations has not been fully elucidated.

In this study, PBMC isolated from healthy Indigenous (Dene or Inuit) and non-Indigenous participants elicited similar levels of pro-inflammatory cytokines TNFα, IL-1β and IL-8, in response to various TLRL representing both bacterial- and viral-associated PAMP. However, there were significant differences in the expression of specific cytokine and innate immune response elements following stimulation of PBMC with bacterial and viral TLRL, between the Indigenous and non-Indigenous participants. These differences were more pronounced between the Dene and non-Indigenous, compared to that between Inuit and non-indigenous participants.

We have previously shown that there is a higher frequency of specific polymorphisms in cytokine-associated genes e.g. IL-6, IL-10, TNF-α and MCP-1 among Indigenous (including the Dene) compared to non-Indigenous populations^9,11^. We have also previously demonstrated specific genetic difference in the killer immunoglobulin-like receptor (KIR) expressed on natural killer (NK) cells predictive of an inhibitory immune profile in Indigenous population, which included the Dene^42^. In this study, we demonstrate this difference functionally wherein TLRL-induced IFN-γ protein, which is predominantly produced by NK cells, is suppressed in PBMC isolated from both Dene and Inuit compared to that in non-Indigenous participants. Similarly, we demonstrate that chemokines that contribute to the recruitment of monocytes and T-cells such as MCP-4, MDC and TARC, and eosinophils e.g. eotaxin, are suppressed in Dene and Inuit compared to non-Indigenous participants. Despite the levels of IFN-γ being suppressed, levels of cytokine IL-12/23 p40 which is an upstream inducer of IFN-γ, was enhanced in PBMC in response to TLRL stimulation in Dene compared to non-Indigenous participants. In addition, we demonstrate that despite our previous studies demonstrating SNPs in TNF-α gene, there is no difference in TNF-α cytokine protein abundance secreted from PBMC in response to different TLRL in Indigenous compared to non-Indigenous participants. In contrast, we show that other inflammatory cytokines e.g. IL-1α and IL-17 levels are elevated in Dene and Inuit respectively compared to non-Indigenous participants. Overall, these results suggest that TLR-induced innate immune responses in PBMC, mediated by TLRL that represent bacterial and viral infections, is differentially expressed in Indigenous compared to non-Indigenous participants.

TLR activation by pathogen-related agonists results in the production of CHDP such as defensins primarily through the TLR-to-NFκB pathway^25^. In addition to being upregulated by TLR activation, CHDP also regulate the TLR-to-NFκB pathway^40,43–45^. Human CHDP are critical elements required for the resolution of infections, as impaired expression of these peptides lead to increased susceptibility to infections^46,47^. Cathelicidins and defensins are the most characterized CHDP in humans. Therefore, we examined the mRNA expression of genes encoding for the human cathelicidin HDP LL-37 *(camp)*, and those encoding for α-defensin (*def-A1*) and β-defensin (*def-B1*), following stimulation of PBMC with various TLRL in this study. We demonstrate that bacterial TLRL MTB lipoprotein and flagellin significantly enhance the expression of *def-A1*, in PBMC isolated from Dene compared to non-Indigenous participants. On the other hand, viral TLRL poly I:C and R848 significantly enhance mRNA expression of *def-B1* in Inuit PBMC compared to that from non-Indigenous participants. However, we were not able to detect the changes in defensins concentrations at the protein level in TC supernatants obtained from PBMC stimulated with the different TLR-ligands. There may be technical reasons for the discrepancy between the mRNA data and the ELISA data for the peptides examined. For example, peptides secreted in the cell culture supernatants may have been degraded or the concentrations may have been below the limit of detection of the ELISA reagents used. However, it also may be that mRNA of the peptides were altered post-transcriptionally affecting either mRNA stability and/or translation^48^. It is known that both α-defensin and β-defensin are functionally critical in the resolution of infection, including TB^49^. Therefore, the observation of enhanced expression of defensin mRNA in response to PAMP including the MTB lipoprotein was counterintuitive. Based on higher expression of defensin genes in Indigenous participants, it may be speculated that these populations can clear infection more effectively. However, it is known that the burden of infections, such as tuberculosis, is higher among Canadian Indigenous populations^50,51^. Therefore, we further examined genetic polymorphisms in the gene encoding for P2X7, a receptor that interacts with defensins to mediate downstream innate immune responses required for resolution of infections.

The human purinergic receptor P2X7 is a transmembrane polypeptide which functions as a ligand gated cation channel. It is highly expressed on hemopoietic immune cells such as macrophages, mast cells, dendritic cells, and lymphoid cells^52,53^. P2X7 is a known receptor for human CHDP α-defensin, β-defensin and LL-37, which mediates antimicrobial immune responses such as the production of cytokines, proliferation and differentiation of immune cells, and apoptosis^40,53–57^. The P2X7 receptor is integrally linked in innate and adaptive immune response^33^; TLR activation by PAMPs results in the release of ATP which in turn activates the P2X7 receptor^34^ and this interplay confers protection against infections^35,58^. Taken together, the TLR-CHDP-P2X7 trifecta is critical in mediating immune response required for the resolution of infections. Indeed, the protective role of P2X7 receptor against pathogens has been demonstrated in animal models^58–61^ and in human infections^62^.

The P2X7 gene is highly polymorphic and contains a large set of SNPs. Genetic association studies have shown that non-synonymous SNPs in P2X7 affect the function of the receptor, and alter the susceptibility of individuals to both infectious and autoimmune diseases, such as TB and rheumatoid arthritis^52,63–71^. Meta-analysis and functional studies of P2X7 SNP (rs3751143) have identified that a polymorphism in the C allele is associated with the susceptibility for MTB^69^. The biochemical effects of the transversion to a mutant CC or AC genotype have been shown to alter the function of the P2X7 receptor. The homozygous recessive CC genotype in P2X7 SNP rs3751143 is associated with the complete loss of function in the receptors, and macrophages with such genotypes fail to clear mycobacterial infections^63^. Macrophages with heterozygous AC genotype in P2X7 SNP rs3751143 result in a 75% reduction in their ability to clear mycobacterial infections^72^. In this study, we demonstrate that the Indigenous (Dene and Inuit) participants have significantly higher percentage of the C allele compared to the non-Indigenous group. We show that the Dene population have significantly more individuals with the CC genotype and C allele frequency out of the three groups examined in this study. A higher frequency of non-functional P2X7 receptors suggest that this could result in an altered CHDP-mediated immune regulation, as it is known that human CHDP α-defensin, β-defensin and LL-37 can induce cytokine responses by interacting with the P2X7 receptor^40,55,73^. Furthermore, a non-functional P2X7 receptor may also result in impaired activation of the receptor by TLR-induced ATP^34,35^ and subsequent impaired activation of the NFκB pathway resulting in suboptimal immune responses required for the control of infections. These corroborate the results in this study, where we have defined variations in cytokine and chemokine production following TLR stimulation in PBMC obtained from Indigenous and non-Indigenous participants.

The results in this study open up new avenue for further examination of the involvement of the P2X7 receptor in anti-infective immunity in Indigenous group. For example, CHDP such as defensins and cathelicidins modulate inflammatory responses including NFκB-induced downstream responses, via the P2X7 receptor^32,40,74,75^. However, other receptors have also been identified for CHDP-mediated immunomodulatory functions^76–78^. As we have shown in this study that certain inflammatory cytokines and chemokines are differentially expressed in PBMC obtained from Indigenous compared to non-Indigenous participants, future experimentation to assess CHDP-induced immunomodulatory responses in PBMC, in the presence and absence of P2X7 antagonists may be valuable. Such future studies will define the relevance of the non-functional P2X7 receptor SNP in anti-infective immunity in Indigenous group.

It should be noted that our study has some limitations; the sample sizes for our study was small therefore our findings might not be generalizable beyond the study groups. We focused our analyses on responses from PBMC and there may be cell type dependent variations that cannot be determined based on this study. A similar study assessing immune responses might have different results if we used other cell or tissue types. Critical analysis of the biomolecular and immunogenic aspects of the immune response and the identification of differences between Indigenous and non-Indigenous Canadians is intended to focus attention on one of the many factors influencing the differential rates of infectious diseases between Canadians. This line of research looks at the variety of immune and genetic responses to infection, driven by evolutionary and environmental factors. Health inequities need to be addressed on multiple fronts so that vaccines and infectious disease treatment regimens consider population-based differences in response to pathogens.

Overall, the results from this study indicate that there are intrinsic differences in TLR-induced immune responses such as expression of specific cytokines, antimicrobial CHDP and associated genetic variations in the P2X7 receptor, between Indigenous compared to non-Indigenous peoples. It is conceivable that these intrinsic genotype and immunomodulatory differences may contribute to the higher susceptibility of infections among Canadian Indigenous populations. A better understanding of the molecular mechanisms that contribute to higher rate of infections among the Canadian Indigenous population will help in the future development of targeted intervention and preventative measures for the control of infection and chronic disease.

## Methods

### Ethics

This study was approved by the University of Manitoba’s Health Research Ethics Board (HS16446). All participants were 18 years or older and provided written informed consent prior to enrolment in the study. The First Nations research principles of Ownership, Control, Access and Possession (OCAP) were followed, and all aspects of the research were shaped, discussed, and approved with the assistance of the Chief and Councils of the First Nations, the Manitoba Inuit Association and approved by the University of Manitoba Health Research Ethics Board^79^.

This research is part of a long-standing partnership with First Nations and Inuit people in Manitoba. The meaning of this research, which is done within this partnership, is understood within a worldview that incorporates both western and Indigenous ways of knowing. This research is grounded in a culture of respect that acknowledges the dignity and equality of all peoples.

### Study participants

Study participants who enrolled in the study to assess TLR-induced responses in PBMC (innate immune response study) were required to provide a blood sample at the University of Manitoba. Dene and Inuit individuals who were visiting or living in Winnipeg were able to enrol in the study. Participants were informed of the study using advertisements, local radio announcements and at community meetings in the Dene First Nation communities in northern Manitoba, and by posters at the Inuit center in Winnipeg. Study participants self-identified as First Nation (Dene, an Indigenous group residing in Manitoba), Inuit (an Indigenous group residing in Manitoba), or non-Indigenous (European-descent residing in Manitoba). Exclusion criteria included history of HIV, tuberculosis or autoimmune diseases, current infection, diabetes, those with immunosuppressive conditions and/or on any medications. Individuals were also excluded if they were a first degree relative of another participant. Seventeen individuals, Dene (n = 5), Inuit (n = 5) and non-Indigenous (n = 7), were enrolled into the study for assessment of TLR-induced response in PBMC.

A total of 114 individuals who self-identified as Dene First Nation, and 99 non-Indigenous individuals were enrolled with informed written consent in the genetic study for the assessment of single nucleotide polymorphisms (SNPs). Individuals who self-identified as Dene First Nation and resided in one of two Dene communities in Manitoba were enrolled in this study. First Nation community consultation determined that convenience sampling, rather than random sampling, was the only methodology considered acceptable for this research. Volunteers for the study responded to posters and radio announcements about the study and a research team met with participants for enrollment. Inclusion in the study required that participants be unrelated (neither first- nor second-degree relatives). Community consultation indicated that conjecture regarding the geographical or evolutionary origins of the participating First Nation peoples was to be avoided. The authors have respected this agreement. Blood samples or buccal swabs were collected in adults and children (with their own and their guardians informed consent). Blood samples were collected from the non-Indigenous individuals enrolled at the University of Manitoba.

### Isolation and culture of human peripheral blood mononuclear cells (PBMC)

Venous blood was collected using BD Vacutainer® (367874) containing sodium heparin as an anticoagulant, in accordance with a protocol approved by the University of Manitoba Ethics Review Board. Blood was diluted 1:1 with complete RPMI1640 medium (Gibco, Invitrogen, Canada), containing 1 mM sodium pyruvate and 10% (v/v) FBS. Human PBMC was separated over Ficoll-Paque^TM^ Premium (GE Healthcare, Buckinghamshire, UK) using SepMate^TM^ tubes (Stemcell Technologies, Vancouver, BC) according to the manufacturer’s instructions. Briefly, Ficoll-Paque^TM^ Premium was pre-layered into the bottom of the SepMate^TM^ tubes through the insert. Diluted blood was carefully pipetted into the SepMate^TM^ tube and centrifuge for 1000 × g for 10 min at room temperature (RT). The top layer containing the enriched PBMC was collected into a new centrifuge tube and washed twice in complete RPMI 1640 medium (300 × g for 10 min). PBMC (1 × 10^6^ cells/mL) were cultured in 6- or 24- well tissue culture plates, rested at 37 °C in a humidified 5% CO_2_ incubator for 1 hour before stimulations with toll-like receptor ligands; MTB lipoprotein (100 ng/ml), poly I:C (1 μg/ml), bacterial lipopolysaccharide (100 ng/ml), flagellin (100 ng/ml), and R848 (1 μg/ml), for 6 h or 24 h as indicated.

### Analyses of cytokines and chemokines using multiplex MesoScale Discovery platform

Human PBMC were stimulated with various TLR ligands, at 37 °C in 5% CO_2_ humidified incubator for 24 h. Cell-free tissue culture (TC) supernatants were used to monitor the production of a panel of 30 human cytokines and chemokines by a V-plex Human Cytokine 30-plex kit (IFN-γ, GMCSF, VEGF-A, IL-2, IL-4, IL-5, IL-6, IL-7, IL-8, IL-8 (HA), IL-10, IL-12 p70, IL-12/23 p40, IL-13, IL-15, IL-16, IL-17α, TNF-α, TNF-β, MIP-1α, MIP-1β, MDC, TARC, IL-1α, IL-1β, Eotaxin, Eotaxin-3, IP-10, MCP-1, MCP-4) using multiplex MesoScale Discovery platform (MesoScale Discovery, Rockville, MD, USA) as per the manufacturer’s instructions. Data was analyzed using the Discovery Workbench 4.0 software (MesoScale Discovery). Multi-Experiment Viewer (MEV) was used to construct the volcano plots and heatmaps. Statistical significances were calculated using the Welch approximation.

### Analyses of CHDP production by ELISA

Human PBMC were stimulated with various TLR ligands, at 37°C in 5% CO_2_ humidified incubator for 6, 24 and 48 h. Concentration of CHDP cathelicidin LL-37, and defensins hBD2 and HNP1-3 was assessed in cell-free TC supernatants by ELISA. LL-37 and HNP1-3 was assessed by ELISA kit obtained from Hycult Biotech (distributed by MJS Biolynx, ON, Canada) and hBD2 ELISA from PeproTech (Montreal, Canada).

### RNA isolation and quantitative real-time PCR (qRT-PCR)

Human PBMC were stimulated for 6 h with various TLR ligands, total RNA was isolated using the Qiagen Miniprep kit and eluted in RNase-free water (Ambion, Thermo Fisher Scientific, Wilmington, DE, USA) as per the manufacturers’ instructions. Total RNA concentration and purity was assessed by NanoDrop 2000 Spectrophotometer (ThermoFisher Scientific, Wilmington, DE, USA). Differential mRNA expression was analyzed using SuperScript III Platinum Two-Step qRT-PCR kit with SYBR Green (Invitrogen, Burlington, ON, Canada) according to the manufacturer’s instructions in the ABI PRISM 7300 Real-Time PCR System. Data analyzed using the 7500 System SDS software (Applied Biosystems, Foster City, CA, USA) and amplicon specificity was determined by melting curve analysis. Fold changes of mRNA expression were calculated after normalization to GAPDH, and using the comparative Ct method^80^. The primers used for qRT-PCR are listed in Table 3.

**Table 3:** Sequence of human primers used for qRT-PCR.

Gene	Forward primer	Reverse primer
*gapdh*	GTCGCTGTTGAAGTCAGAGG	GAAACTGTGGCGTGATGG
*defa1*	CCTGCCTAGCTAGAGGATCTGT	CATCAGCTCTTGCCTGGAGT
*defb1*	TGTCTGAGATGGCCTCAGGT	GGGCAGGCAGAATAGAGACA
*camp*	TCGGATGCTAACCTCTACCG	ACAGGCTTTGGCGTGTCT

### DNA isolation and polymerase chain reaction-restriction fragment length polymorphism (PCR-RFLP)

Venous blood was collected from volunteers using BD Vacutainer® with K_2_EDTA as anticoagulant, in accordance with a protocol approved by the University of Manitoba Ethics Review Board. Buccal samples were collected using Cap-Shure sterile cotton tipped applicators with aerated tip protector Puritan, United Kingdom). DNA was isolated using QIAamp DNA Mini Kit (Qiagen, U.S.A.), and amplified with REPLI-g Mini Kit (QIAGEN, U.S.A.) according to the manufacturer’s instructions.

**Figure 7:**
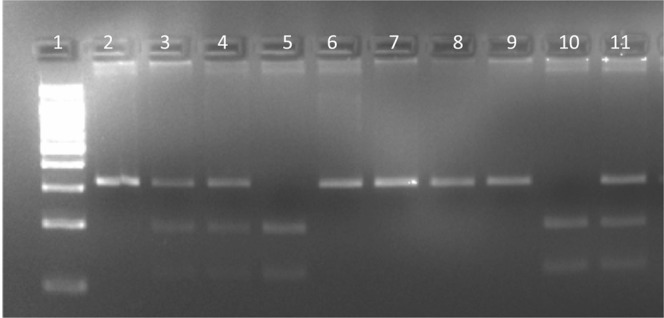
P2X7 Restriction fragment length polymorphism (RFLP). The RFLP digest was separated and visualized on a 3% agarose gel stained with ethidium bromide at 120 volts for 30 minutes with the expected band lengths of A (317) and C (118, 199). Ladder (100 base pair ladder (Lane 1), A/A genotype (Lanes 1, 6–9 and 12–19), A/C (Lanes 3, 4, 11), C/C genotype (Lanes 5 and 10) (see Supplementary Fig. S3 for original image).

PCR-RFLP analysis was used to genotype P2X7 rs3751143. The primers used for PCR-RFLP are listed in Table 4. Platinum Taq High Fidelity 500 Reactions PCR kit was used for PCR amplification and performed using a 25 uL mixture according to the Life Technologies Real-time PCR instruction manual. Briefly, the PCR cycling parameters were as followed: step 1) 94 °C for 300 seconds, step 2) 94 °C for 30 seconds, step 3) 54 °C for 30 seconds, step 4) 72 °C for 45 seconds, step 5) repeat steps 2–4 for 30 cycles, step 6) 72 °C for 300 seconds. PCR was then visualized on a 2.5% agarose gel stained with ethidium bromide at 120 volts for 30 minutes. The PCR amplified mixture underwent RFLP analysis and was digest with the restriction enzyme HaeII (New England BioLabs, U.S.A)^51^. The RFLP digest was separated and visualized on a 3% agarose gel stained with ethidium bromide at 120 volts for 30 minutes with the expected band lengths of A (317) and C (118, 199) (Fig. 7).

**Table 4:** Sequence of human P2X7 rs3751143 primers used for PCR-RFLP.

P2X7 rs3751143	Sequence (5′-3′)
Forward	AGACCTGCGATGGACTTCACAG
Reverse	AGCGCCAGCAAGGGCTC

### Statistical analysis

Statistical significance for relative mRNA expression determined by qRT-PCR was assessed using Mann-Whitney U test, and that for cytokine/chemokine production obtained with the MSD platform was assessed using the Welch approximation. A p-value of less than 0.05 was considered to be statistically significant. The allele and genotype frequencies for Indigenous and the non-Indigenous populations were compared using the Pearson chi squared tests to determine assess the extent of differences. Hardy-Weinberg Equilibrium were determined via Pearson’s chi-square test.

## Supplementary information

Supplementary Figures
