# Supplementary material for: Polymorphisms in the P2X7 receptor, and differential expression of Toll-like receptor-mediated cytokines and defensins, in a Canadian Indigenous group
Source: Sci Rep. 2019 Oct 2;9:14204. doi: 10.1038/s41598-019-50596-0 (PMC6775093; doi:10.1038/s41598-019-50596-0)

Polymorphisms in the P2X7 receptor and differential expression of Toll-like receptor-mediated cytokines and defensins in a Canadian Indigenous group.

Authors: Catlin Semple[1+], Ka-Yee Grace Choi[2+], Andrea Kroeker[2+], Lizette Denechezhe[3], Pamela Orr[1,2,4], Neeloffer Mookherjee[1,5,#], Linda Larcombe[1,2,4,*#]

[1]Department of Medical Microbiology and Infectious Disease, Max Rady Faculty of Medicine, University of Manitoba, Winnipeg, MB, Canada.

[2]Department of Internal Medicine, Max Rady Faculty of Medicine, University of Manitoba, Winnipeg, MB, Canada.

[3]Northlands Denesuline First Nation, MB, Canada.

[4]Department of Community Health Sciences, Max Rady Faculty of Medicine, University of Manitoba, Winnipeg, MB, Canada.

[5]Department of Immunology, Max Rady Faculty of Medicine, University of Manitoba, Winnipeg, MB, Canada.

***Corresponding author:**

Linda Larcombe
531A Basic Medical Sciences Building, 745 Bannatyne Ave, Winnipeg, MB, R3E 0J9, Canada.
Phone: 204-975-7726. Email: linda.larcombe@umanitoba.ca

Supplementary Figure S1: mRNA expression of gene encoding for cathelicidin LL-37 in PBMC isolated from Indigenous and Non-Indigenous participants. Human PBMC isolated from the study participants were stimulated with different TLR ligands; MTB lipoprotein (100ng/ml), LPS (100 ng/ml), Flagellin (100 ng/ml) or R848 (1 µg/ml), for 6 hr. mRNA isolated was examined for the expression of *camp* by quantitative real-time PCR. Relative fold changes were calculated compared to the expression in the unstimulated cells normalized to 1, using the standard ΔΔCt method, after normalization with 18sRNA expression. Each dot represents an independent experiment from PBMC isolated independent participants. Mann-Whitney U test was used for statistical analyses.

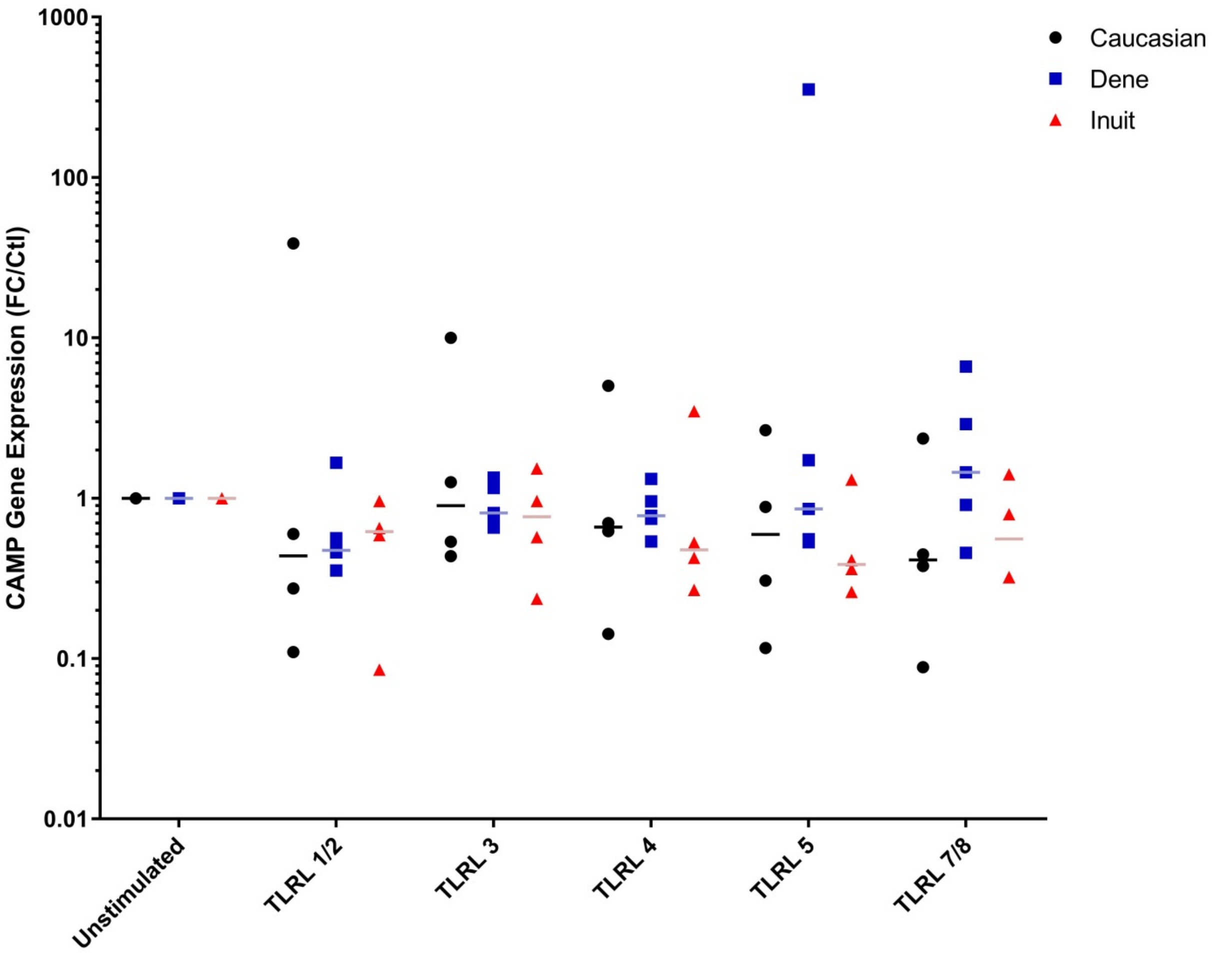

Supplementary Figure S2: Production of hBD2, HNP1-3 and LL-37 monitored by ELISA. Human PBMC isolated from the study participants were stimulated with different TLR ligands; MTB lipoprotein (100ng/ml), LPS (100 ng/ml), Flagellin (100 ng/ml) or R848 (1 μg/ml) for 24 h. Tissue culture supernatants were examined for the production of **(A)** cathelicidin LL-37, and defensins **(B)** HNP1-3 and **(C)** hBD2 by ELISA. Each dot represents an independent experiment from PBMC isolated independent participants. Graph shows data for Caucasian and Dene participants. Mann-Whitney U test was used for statistical analyses.

A.

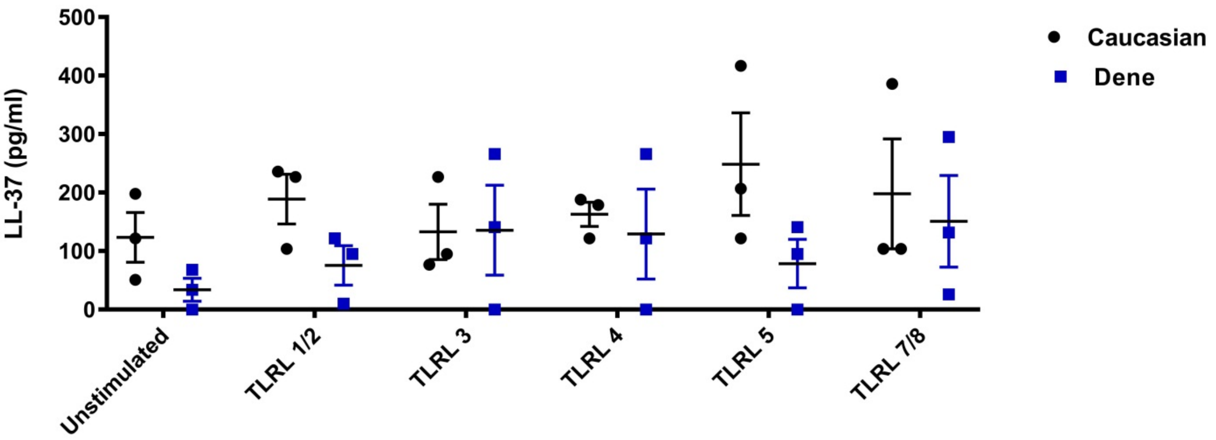

B.

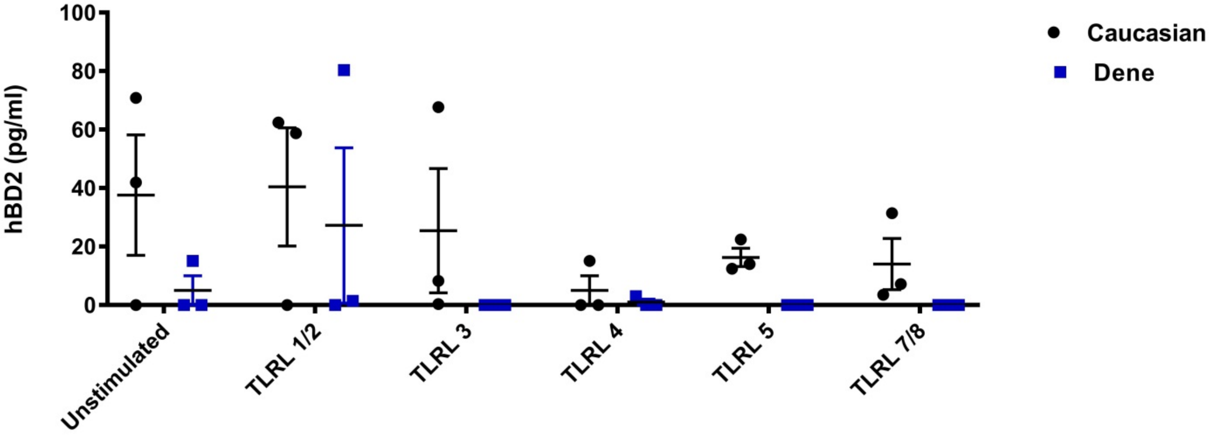

C.

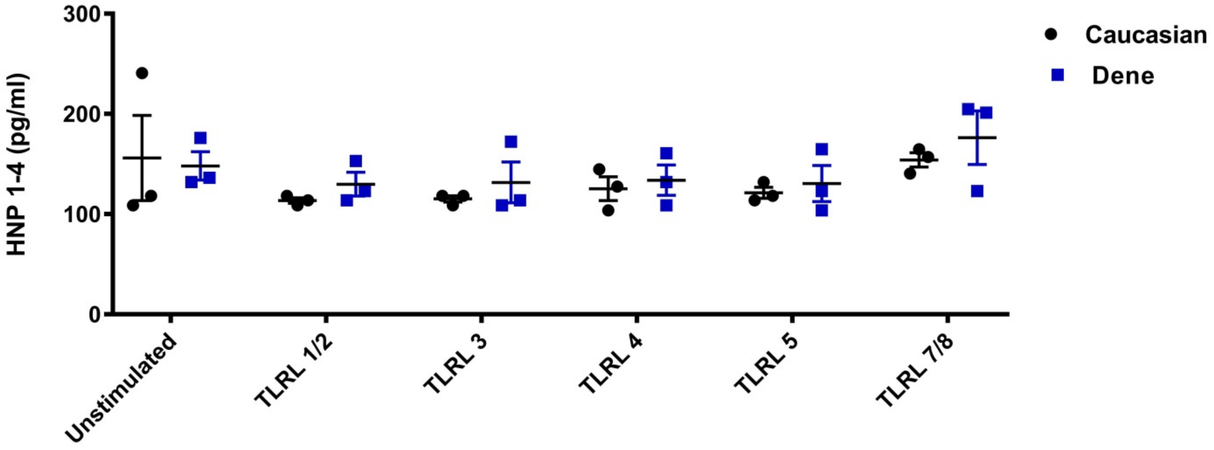

300
200
100
0
HNP 1-4 (pg/ml)
Unstimulated
TLRL 1/2
TLRL 3
TLRL 4
TLRL 5
TLRL 7/8
Caucasian
Dene

Supplementary Figure S3. Original full-size image of restriction fragment length polymorphism (RFLP) results on a 3% agarose gel for P2X7. The RFLP digest was separated and visualized on a 3% agarose gel stained with ethidium bromide at 120 volts for 30 minutes with the expected band lengths of A (317) and C (118, 199). Ladder (100 base pair ladder (Lane 1), A/A genotype (Lanes 1, 6-9 and 12-19), A/C (Lanes 3, 4, 11), C/C genotype (Lanes 5 and 10), and the negative control (Lane 20). Area cropped for creating Figure 7 is shown in the white rectangle.

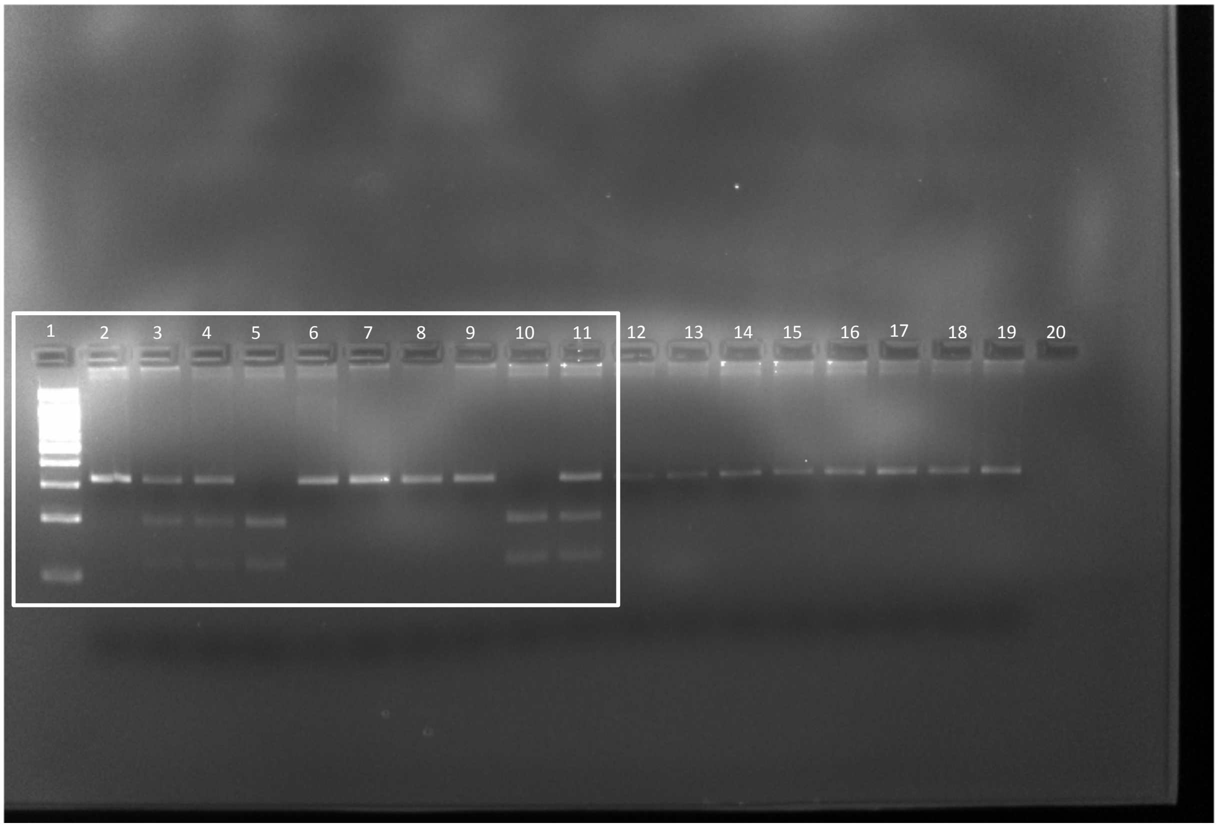

Supplement: Supplementary file 1 — Supplementary Figures [file 41598_2019_50596_MOESM1_ESM.pdf]
